# Sex and β-Endorphin Influence the Effects of Ethanol on Limbic *Gabra2* Expression in a Mouse Binge Drinking Model

**DOI:** 10.3389/fgene.2018.00567

**Published:** 2018-11-29

**Authors:** Erin M. Rhinehart, Todd B. Nentwig, Diane E. Wilson, Kiarah T. Leonard, Bernie N. Chaney, Judith E. Grisel

**Affiliations:** ^1^Department of Biology, Susquehanna University, Selinsgrove, PA, United States; ^2^Department of Psychology, Neuroscience Program, Bucknell University, Lewisburg, PA, United States

**Keywords:** alcohol, BNST, CeA, GABA_A_, sex differences, stress, POMC

## Abstract

Binge drinking is a widespread problem linked to increased risk for alcohol-related complications, including development of alcohol use disorders. In the last decade, binge drinking has increased significantly, specifically in women. Clinically, sexually dimorphic effects of alcohol are well-characterized, however, the underlying mechanisms for these dimorphisms in the physiological and behavioral effects of alcohol are poorly understood. Among its many effects, alcohol consumption reduces anxiety via the inhibitory neurotransmitter GABA, most likely acting upon receptors containing the α-2 subunit (*Gabra2*). Previous research from our laboratory indicates that female mice lacking the endogenous opioid peptide β-endorphin (βE) have an overactive stress axis and enhanced anxiety-like phenotype, coupled with increased binge-like alcohol consumption. Because βE works via GABA signaling to reduce anxiety, we sought to determine whether sexually dimorphic binge drinking behavior in βE deficient mice is coupled with differences in CNS *Gabra2* expression. To test this hypothesis, we used βE knock-out mice in a “drinking in the dark” model where adult male and female C57BL/6J controls (βE +/+) and βE deficient (βE -/-; B6.129S2-Pomc^tm1Low^/J) mice were provided with one bottle of 20% ethanol (EtOH) and one of water (EtOH drinkers) or two bottles of water (water drinkers) 3 h into the dark cycle for four consecutive days. Following a binge test on day 4, limbic tissue was collected and frozen for subsequent qRT-PCR analysis of *Gabra2* mRNA expression. Water-drinking βE +/+ females expressed more *Gabra2* in central nucleus of the amygdala and the bed nucleus of the stria terminalis than males, but this sex difference was absent in the βE -/- mice. Genotype alone had no effect on alcohol consumption or drug-induced increase in *Gabra2* expression. In contrast, βE expression had bi-directional effects in females: in wildtypes, *Gabra2* mRNA was reduced by binge EtOH consumption, while EtOH increased expression in βE -/- females to levels commensurate with drug-naïve βE +/+ females. These results support the contention that βE plays a role in sexually dimorphic binge-like EtOH consumption, perhaps through differential expression of GABA_A_ α2 subunits in limbic structures known to play key roles in the regulation of stress and anxiety.

## Introduction

At least 10 million Americans have an alcohol use disorder (AUD), making alcohol one of the most abused drugs in the United States (Substance Abuse and Mental Health Services, 2018). According to epidemiological data, more men than women have AUDs; however, the gap between the rates of AUD in men and women is rapidly closing (Keyes et al., 2011; Gowing et al., 2015; Grant et al., 2017; Substance Abuse and Mental Health Services, 2018). In fact, male and female teenagers, aged 12–17, have equivalent alcohol usage rates (Gowing et al., 2015; Becker et al., 2017; Grant et al., 2017; Substance Abuse and Mental Health Services, 2018), with females exhibiting a disconcertingly rapid increase in binge drinking behavior (Jennison, 2004; Dwyer-Lindgren et al., 2015; Gowing et al., 2015; Becker et al., 2017; Grant et al., 2017; Substance Abuse and Mental Health Services, 2018). AUDs have complex etiologies with a strong genetic component (Gelernter and Kranzler, 2009). The changing sociocultural landscape has undoubtedly contributed to the escalating incidence of AUD in females (for example: da Mata Ribeiro et al., 2014; Glantz et al., 2014), but females who begin drinking alcohol may have greater vulnerability to AUD due to a variety of biological factors, such as sexually dimorphic gene expression in the brain (for review see: Becker et al., 2017). While historically fewer females than males experiment with drugs and alcohol, when females do imbibe they progress to addiction more often and more quickly than males (Piazza et al., 1989; Keyes et al., 2010; Valentino and Bangasser, 2016; Becker et al., 2017). This sexually dimorphic “telescoping” phenomenon, frequently observed in women is likely to be at least partly rooted in biological factors (Marinelli et al., 2003; Svikis et al., 2006; Rajasingh et al., 2007; Satta et al., 2018). In accordance, greater voluntary alcohol intake in females has been reported in multiple species (Forger and Morin, 1982; Morin and Forger, 1982; Li and Lumeng, 1984; Tambour et al., 2008) supporting the notion that females may possess greater vulnerability to alcohol addiction (Lynch, 2006).

There are a wide variety of potential explanations for sex differences in AUD vulnerability. For example, sexual dimorphisms in stress reactivity and stress-related disorders (Breslau et al., 1998; Bangasser et al., 2010; Hartwell and Ray, 2013; Bangasser and Valentino, 2014; Bandelow and Michaelis, 2015) could be partly responsible (Lynch, 2006). A preponderance of evidence indicates that stress-related psychiatric disorders, such as anxiety and post-traumatic stress disorder, occur more frequently in women than men (Breslau et al., 1998; Bangasser et al., 2010; Hartwell and Ray, 2013; Bangasser and Valentino, 2014; Bandelow and Michaelis, 2015). Sexual dimorphisms in the incidence of stress-related disorders are partly related to gender differences in psychological affect, social role identification and other sociocultural factors, but a significant sex disparity remains even after the contribution of these variables has been removed (Kendler et al., 1995a,b; Breslau et al., 1998; Tolin and Foa, 2006). In addition, AUD is frequently comorbid with anxiety disorders (Cullen et al., 2013; Gilpin and Weiner, 2017), providing additional evidence of a connection between AUD and stress-related disorders. In humans and other species, stress increases vulnerability to addiction, and it is an intrinsic driver of alcohol use and relapse (McGonigle et al., 2016; Clay et al., 2018; Milivojevic and Sinha, 2018). In addition, the anxiolytic properties of alcohol make it viable as a potential stress-coping strategy (Watt et al., 2014; Bos et al., 2016; McGonigle et al., 2016; Gorka and Shankman, 2017). Interestingly, females are more likely to drink alcohol to alleviate a negative emotional state, like that induced by chronic stress (Adams et al., 1991; Erol and Karpyak, 2015; Karpyak et al., 2016), and females are more susceptible to stress-induced drinking behaviors (Gorka et al., 2012; McGonigle et al., 2016). Therefore, it is critical to understand the influence of stress-reactivity on the mechanisms underlying sex differences in addiction, especially as the incidence of stress-related disorders continues to increase (Grucza et al., 2008; Thorisdottir et al., 2017).

When functioning properly, behavioral and physiological stress responses are adaptive. An adaptive stress response is limited in duration and followed by the restoration of homeostasis. Acute stress activates the hypothalamic-pituitary-adrenal (HPA) axis, stimulating corticotropin-releasing hormone (CRH) secretion from the paraventricular nucleus (PVN) of the hypothalamus and a subsequent increase in the precursor protein, proopiomelanocortin (POMC). POMC is then proteolytically cleaved into several signaling peptides, including the endogenous opioid peptide, β-endorphin (βE), an opioid agonist with high affinity for μ- and δ-opioid receptors. βE provides negative feedback to limit the duration of HPA axis activation, and it acts within the amygdala (AMY) to regulate behavioral responses to stressful stimuli and restore homeostasis (Charmandari et al., 2005). Genetic conditions that result in a reduction or elimination of βE signaling lead to an overactive HPA axis and an inability to exhibit adaptive coping responses to stress (Grisel et al., 2008; Barfield et al., 2010; McGonigle et al., 2016; Nentwig et al., 2018). In general, there is an inverse relationship between βE and anxiety-like behavior in mice (Grisel et al., 2008; Barfield et al., 2010; Nentwig et al., 2018), and lack of βE induces hyperactivity of the HPA axis (McGonigle et al., 2016; Nentwig et al., 2018). Therefore, genetic variability in the βE system may underlie stress-related disease vulnerability, which would impact the risk of AUD.

Because alcohol is frequently used for anxiolytic purposes, innate hyper-reactivity to stress increases addiction vulnerability (Sinha, 2001; Stephens and Wand, 2012; Blaine and Sinha, 2017). The anxiolytic effects of alcohol are mediated by the inhibitory neurotransmitter, gamma-amino butyric acid (GABA) (Engin et al., 2012; Lindemeyer et al., 2017). Alcohol potentiates GABA signaling at the GABA_A_ receptor (GABA_A_R) in limbic regions of the brain such as the ventral tegmental area (VTA), nucleus accumbens (NAc), central nucleus of the amygdala (CeA), and bed nucleus of the stria terminalis (BNST) (Suzdak et al., 1986; Hyytia and Koob, 1995; Xiao and Ye, 2008; Guan and Ye, 2010; Melon and Boehm, 2011). The GABA_A_R is a heterogeneous pentameric, transmembrane chloride ion channel, and the subunit composition of this receptor determines the pharmacological properties of the receptor (Barnard et al., 1998; Hevers and Luddens, 1998; Boehm et al., 2004; Olsen and Sieghart, 2009). The gene for the α2 (GABA_A_α2) subunit of the GABA_A_R is highly connected with vulnerability to addiction in humans (Haughey et al., 2008; Enoch et al., 2009; Bierut et al., 2010; Enoch et al., 2012). The GABA_A_α2 gene (*Gabra2*) is expressed in the AMY, NAc, VTA, BNST, cortex, thalamus, and hypothalamus (Herbison and Fenelon, 1995; Schwarzer et al., 2001; Boehm et al., 2004; Dixon et al., 2010), with 15–20% of all GABAAR in the brain containing the GABAAα2 subunit (Pirker et al., 2000; Engin et al., 2012). Mice with a mutation in the Gabra2 gene have heightened baseline levels of anxiety (Dixon et al., 2008; Vollenweider Smith et al., 2011; Engin et al., 2012). Moreover, single nucleotide polymorphisms (SNPs) in the Gabra2 gene are robustly related to increased risk for AUDs (Edenberg et al., 2004; Enoch et al., 2006, 2012; Haughey et al., 2008; Bierut et al., 2010; Engin et al., 2012; Ittiwut et al., 2012; Uhart et al., 2013; Kuperman et al., 2017). Therefore, α2-containing GABA_A_Rs represent a potential link between sexually dimorphic stress-related disorders and AUD vulnerability.

A variety of previous studies have used animal models to attempt to elucidate the mechanisms underlying sex differences in EtOH-related behavior to shed light on the increasing female incidence of AUD. Data from rodent studies support the notion that females exposed to alcohol will imbibe more than males and become dependent more quickly than males (Li and Lumeng, 1984; Adams et al., 1991). Previous data from our laboratory indicate that βE is integral in the sexually dimorphic connection between stress and EtOH consumption (Barfield et al., 2010; McGonigle et al., 2016; Nentwig et al., 2018). For example, βE deficient mice have enhanced stress reactivity and anxiety-like behaviors as well as a decreased ability to behaviorally manage stress (Grisel et al., 2008; Barfield et al., 2010; McGonigle et al., 2016; Nentwig et al., 2018). These phenotypic differences are accompanied by greater CRH expression in the hypothalamus, AMY and BNST, which is correlated with increased serum cortisol and hypertrophied adrenal glands (McGonigle et al., 2016). Previous studies have also shown that stressed female βE deficient animals exhibit greater alcohol consumption (McGonigle et al., 2016), possibly in an effort to normalize HPA axis hyperactivity. Using the drinking in the dark (DID) paradigm, our laboratory also has also shown that female mice deficient for βE can use binge drinking behavior to normalize cortisol levels and decrease CRH expression in the BNST and CeA (Nentwig et al., 2018). These data provide support for the interaction of sex, βE and the stress axis in the behavioral regulation of EtOH consumption. The underlying molecular substrates of this interaction are currently unknown. Given the connection between AUD, stress, and the *Gabra2* gene outlined above, we tested the hypothesis that βE deficiency correlates with sexually dimorphic differences in *Gabra2* gene expression in the limbic system.

## Materials and Methods

### Animals

Adult male and female C57BL/6J (βE +/+) and B6.129S2-Pomc^tm1Low^/J (βE -/-) mice were either bred in-house and weaned at 21 days from stock obtained from Jackson Laboratories (Bar Harbor, ME, United States) or purchased as adults from Jackson Laboratories in which case they were acclimated at least 10 days prior to the onset of any experimental procedures. The βE -/- mice were developed in the laboratory of Malcolm Low and are fully backcrossed onto a C57BL/6J background. Transgenic mice harbor a truncated *Pomc* transgene that prevents synthesis of βE, although other POMC protein products remain unchanged, such that homozygotes cannot synthesize βE and heterozygotes produce ∼50% of wildtype levels (Rubinstein et al., 1996). βE -/- males have been shown to exhibit an overweight phenotype that increases with age, although we observed no differences in weight across genotypes of either sex in the present study. Mice were group-housed by sex and genotype before the start of the experiment, and individually during the experiment, in Plexiglas^®^ cages with corncob bedding and *ad libitum* access to chow and water. The animal colony and experimental room were maintained at ∼21°C with a 12-h/12-h reverse light/dark cycle (lights off at 0930). We assessed mRNA expression from brains harvested in animals used in a previous study (Nentwig et al., 2018) and evaluated a separate group of naïve subjects in the DID protocol. Procedures were in accordance with the National Institute of Health guidelines and approved by the Bucknell University Institutional Animal Care and Use Committee.

### Drinking in the Dark (DID) Procedures

A 2-bottle, 4-day DID procedure was performed as described previously (Nentwig et al., 2018) with water continuously available in one bottle for all mice. Mice were acclimated to individual housing for at least 7 days prior to the 4-day DID testing. On days 1–3 of DID testing, for 2 h beginning 3 h into the dark cycle, mice had access to two 25 mL graduated cylinders containing either 20% EtOH in tap water (v/v) or tap water alone (EtOH drinkers), while control groups received tap water in both bottles (water drinkers). On day 4, access to EtOH or the additional water tube was extended to a 4 h binge test session. Fluid intake levels were measured by a trained observer blind to experimental condition by reading gradations on bottles with accuracy to the nearest 0.1 mL.

### Brain Punch Protocol and qRT-PCR

Immediately following the 4 h binge test on day 4, subjects were individually transported to an adjacent room, anesthetized using isoflurane, and rapidly decapitated. Brains were removed, frozen on dry ice, and stored at -80°C for gene expression using qRT-PCR. Frozen tissue was sliced on a Thermo Fisher HM 550 cryostat (Thermo Fisher Scientific, Waltham, MA, United States) and bilateral 1.5 mm cylindrical punches were taken of the NAc (+1.94 to +0.86 mm, with respect to bregma), BNST (+0.62 to -0.22 mm), and CeA (-0.82 to -1.82 mm) and immediately submerged in QIAzol lysis buffer (Qiagen GmbH, Hilden, Germany). Each sample tube containing one brain region from one mouse was homogenized immediately after sectioning. Total RNA was extracted using the Qiagen RNeasy Lipid Tissue Minikit (Qiagen GmbH, Hilden, Germany) according to manufacturer’s instructions. Concentration and purity of eluted RNA was verified using the NanoDrop Lite UV spectrophotometer (Thermo Fisher Scientific, Waltham, MA, United States) and 500 ng of total RNA was reverse-transcribed using the iScript^TM^ cDNA Synthesis Kit (BioRad, Hercules, CA, United States) also according to manufacturer’s instructions. qRT-PCR was performed using FastStart Essential DNA Probes Master Mix (Roche Diagnostics, Indianapolis, IN, United States) according to manufacturer’s instructions. PrimeTime^®^ XL qRT-PCR Assays designed by IDT (Integrated DNA Technologies, Coralville, IA, United States) were performed in duplicate on a LightCycler 96 (Roche Diagnostics, Indianapolis, IN, United States). All assays had similar optimum PCR efficiencies. For all qRT-PCR experiments, GAPDH gene expression was used as the reference gene and relative changes in gene expression were illustrated using the 2^-ΔΔCT^ method (Schmittgen and Livak, 2008).

### Statistical Analysis

EtOH consumption and preference were calculated daily. From these we determined the average intake per 2 h period, the average preference across the 4-day procedure and the intake during the 4 h binge test. Two-way ANOVAs with genotype and treatment (EtOH drinkers vs. water drinkers) as factors were used to analyze EtOH consumption and preference as well as *Gabra2* mRNA expression in the NAc, BNST, VTA, and CeA. Statistical analyses for the mRNA expression were conducted on raw data before transformation using the 2^-ΔΔCT^ method. Bonferroni *post hoc* tests were used to correct for multiple comparisons following significant main effects and interactions. Degrees of freedom may differ between groups/brain regions due to unquantifiable tissue. Drinking data were analyzed using SPSS 24.0 software while GraphPad Prism 7.0 was used to assess differences in gene expression between groups. Data are presented as mean ± SEM. Effects were considered statistically significant at *p* ≤ 0.05.

## Results

### Female Mice Lacking βE Exhibit Enhanced Proclivity for Binge-Like Alcohol Consumption

**FIGURE 1:**
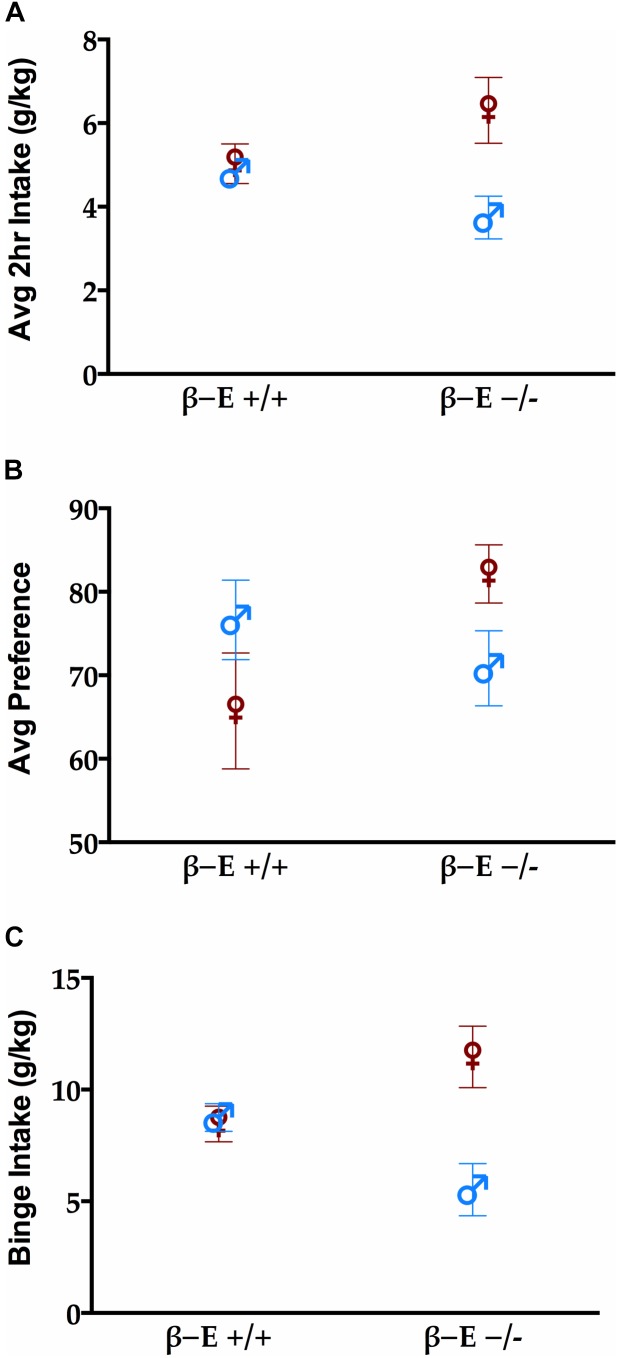
βE masks sex differences in binge-like EtOH consumption. **(A)** Average 2 h intake across 4 day drinking in the dark (DID). **(B)** Preference for EtOH solution during the 4 h binge test. **(C)** Consumption of EtOH during the 4 h binge test on day 4 of the DID procedure. A two-way ANOVA revealed a main effect of sex (female mice > male mice) and a sex by genotype interaction. Post hoc analyses indicated that the βE –/– female mice consumed more EtOH than βE –/– male and βE +/+ female mice. Data are presented as means ± SEM.

Replicating previous results (Nentwig et al., 2018), we found that the absence of βE resulted in sex differences in drinking behavior. Figure 1A shows the average 2 h intake across all 4 days of the DID procedure in each group. There was a main effect of sex [*F*_(1,28)_ = 6.73, *p* < 0.05] but not genotype [*F*_(1,28)_ = 0.041, *p* > 0.05]. There was a significant interaction between sex and genotype reflecting the fact that female βE -/- mice consumed more than other groups [*F*_(1,28)_ = 4.736, *p* < 0.05]. There were neither sex nor strain differences in preference for EtOH [*F*_(1,28)_ = 0.001 and 1.087, respectively, both *p* > 0.05], however, there was a significant interaction between sex and genotype for EtOH preference [*F*_(1,28)_ = 4.772, *p* < 0.05] (Figure 1B). Finally, during the 4 h binge test (Figure 1C), there was a main effect of sex [*F*_(1,28)_ = 7.426, *p* < 0.05], but not genotype [*F*_(1,28)_ = 0.013, *p* > 0.05], and again a significant interaction between sex and genotype [*F*_(1,28)_ = 8.983, *p* < 0.05]. To follow up on the significant interaction suggesting that for females absent βE increased alcohol preference and consumption while the opposite was true for males (deficiency decreased drinking) simple effects of genotype were evaluated within each sex. After Bonferroni correction (i.e., alpha set at 0.025) none of these comparisons reached significance, indicating that the interactive effects of genotype and sex support a moderate bi-directional influence of βE on behavior (*p*’s for females: 0.186, 0.053, and 0.082 for average g/kg, average preference, and binge consumption; and for males the analogous values were 0.078, 0.390, and 0.029).

### In Female Mice, the Effects of EtOH on *Gabra2* Gene Expression Depend Upon βE

**FIGURE 2:**
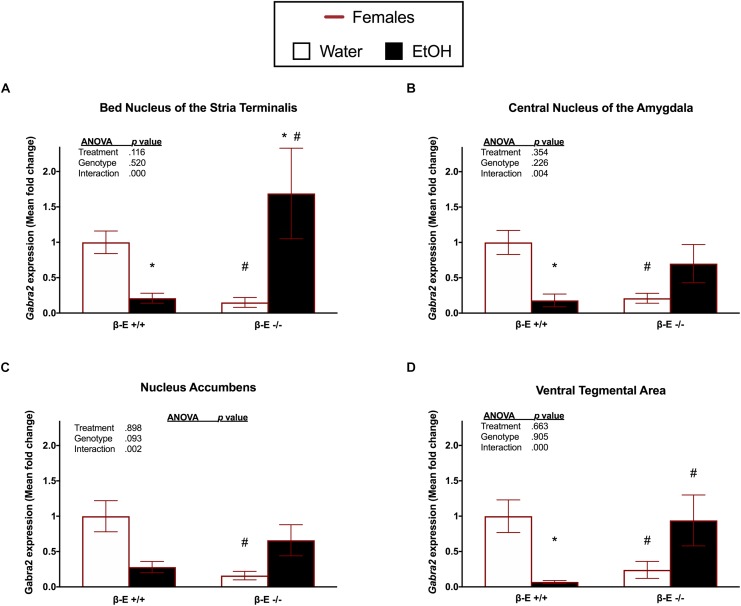
Effects of ethanol on *Gabra2* gene expression in stress- and reward-related brain regions of βE +/+ and βE –/– female mice. *Gabra2* mRNA expression following binge-like consumption of either EtOH and water or water only in the DID paradigm. Two-way ANOVAs were used to examine the main and interaction effects of genotype (βE +/+, βE –/–) and treatment (EtOH drinker, water drinkers) in each brain region, results of which are depicted within each graph. **(A)** In the BNST, *post hoc* analysis indicated that, under basal conditions (water drinkers), βE –/– females have less *Gabra2* expression, relative to βE +/+ females. Further, EtOH consumption reduced *Gabra2* expression in βE +/+ females, but increased expression in βE –/– females, such that βE –/– females exhibited higher *Gabra2* expression than βE +/+ females who engaged in binge-like EtOH consumption. **(B)** In the CeA, *post hoc* analysis indicated that, under basal conditions, βE –/– females have lower *Gabra2* expression, relative to βE +/+ females. Similar to the BNST, EtOH also reduced *Gabra2* expression in the CeA of βE +/+ females. **(C)** In the NAc, *post hoc* analysis indicated that, under basal conditions, βE –/– females have lower *Gabra2* expression, relative to βE +/+ females. **(D)** In the VTA, *post hoc* analysis indicated that, under basal conditions, βE –/– females have lower *Gabra2* expression, relative to βE +/+ females. Following EtOH consumption, βE +/+ females exhibited lower *Gabra2* expression than EtOH-drinking βE –/– females due to an EtOH-induced reduction in *Gabra2* in βE +/+ females. ^∗^*p* < 0.05 compared with the water drinkers within the same genotype and ^#^*p* < 0.05 compared with the βE +/+ genotype group that received the same treatment. Data are presented as means ± SEM; Bonferroni correction for multiple comparisons.

To determine if differential expression of the *Gabra2* gene is involved in the mechanism underlying the sexually dimorphic effects of βE expression on binge-like EtOH consumption, we used qRT-PCR to analyze *Gabra2* gene expression in the BNST, CeA, NAc, and VTA of male and female βE +/+ and βE -/- mice. Two-way ANOVAs on *Gabra2* expression were performed for each brain region and they all yielded significant genotype by treatment interactions [BNST: [*F*_(1,20)_ = 30.637, *p* < 0.001], CeA: [*F*_(1,25)_ = 9.963, *p* = 0.004], NAc: [*F*_(1,22)_ = 11.931, *p* = 0.002], VTA: [*F*_(1,21)_ = 17.936, *p* < 0.001]], but no main effects of genotype [BNST: [*F*_(1,20)_ = 0.429, *p* = 0.520], CeA: [*F*_(1,25)_ = 1.539, *p* = 0.226], NAc: [*F*_(1,22)_ = 3.079, *p* = 0.093], VTA: [*F*_(1,21)_ = 0.015, *p* = 0.905]] or treatment [BNST: [*F*_(1,20)_ = 2.700, *p* = 0.116], CeA: [*F*_(1,25)_ = 0.891, *p* = 0.354], NAc: [*F*_(1,22)_ = 0.017, *p* = 0.898], VTA: [*F*_(1,21)_ = 0.196, *p* = 0.663]]. *Post hoc* analysis following the BNST genotype by treatment interaction indicated that, under basal conditions (water drinkers), βE -/- females have lower *Gabra2* expression, relative to βE +/+ females (*p* < 0.05). Further, EtOH consumption reduced *Gabra2* expression in βE +/+ females (*p* < 0.05), but increased expression in βE -/-females (*p* < 0.05), such that βE -/- females exhibited higher *Gabra2* expression than βE +/+ females who engage in binge-like EtOH consumption (*p* < 0.05; Figure 2A). *Post hoc* analysis following the CeA genotype by treatment interaction indicated that, under basal conditions, βE -/- females have lower *Gabra2* expression, relative to βE +/+ females (*p* < 0.05). Similar to the BNST, EtOH also reduced *Gabra2* expression in the CeA of βE +/+ females (*p* < 0.05; Figure 2B). *Post hoc* analysis following the NAc genotype by treatment interaction indicated that, under basal conditions, βE -/- females have lower *Gabra2* expression, relative to βE +/+ females (*p* < 0.05; Figure 2C). *Post hoc* analysis following the VTA genotype by treatment interaction indicated that, under basal conditions, βE -/- females have lower *Gabra2* expression, relative to βE +/+ females (*p* < 0.05). Following EtOH consumption, βE +/+ females exhibited lower *Gabra2* expression than EtOH-consuming βE -/- females (*p* < 0.05) due to an EtOH-induced reduction in *Gabra2* in βE +/+ females (*p* < 0.05; Figure 2D).

### βE Expression Has No Effect on *Gabra2* Gene Expression in Limbic Brain Areas of Male Mice

**FIGURE 3:**
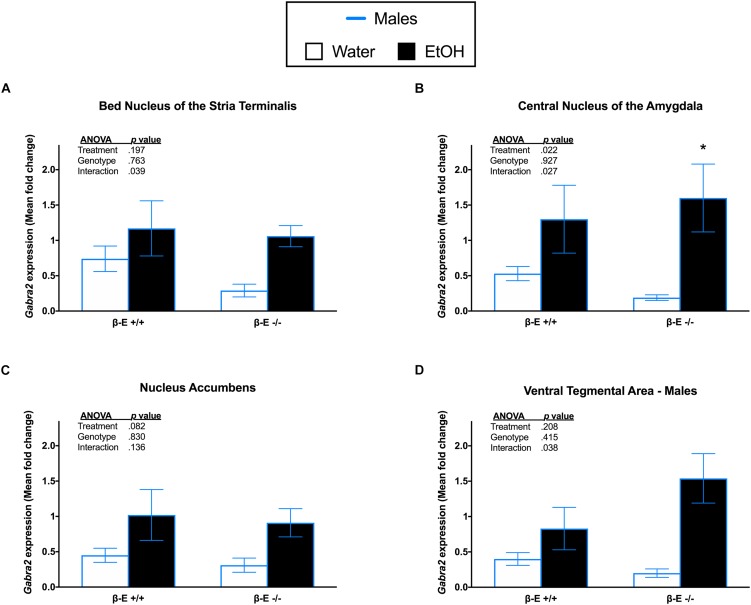
Effects of ethanol on *Gabra2* gene expression in stress- and reward-related brains regions of βE +/+ and βE –/– male mice. *Gabra2* mRNA expression following binge-like consumption of either EtOH and water or water only in the DID paradigm. Two-way ANOVAs were used to examine the main and interaction effects of genotype (βE +/+, βE –/–) and treatment (EtOH drinker, water drinkers) in each brain region, results of which are depicted within each graph. **(A)** In the BNST, there was a significant interaction, but *post hoc* analysis did not reveal any significant differences between groups. **(B)** In the CeA, *post hoc* analysis indicated that binge-like EtOH consumption increased *Gabra2* expression in βE –/– males. **(C)** In the NAc, there was a significant interaction, but *post hoc* analysis revealed no significant differences between groups. **(D)** Similar to the NAc, in the VTA there was a significant interaction, but *post hoc* analysis revealed no significant differences between groups. ^∗^*p* < 0.05 compared with the water group within the same genotype. Data are presented as means ± SEM; Bonferroni correction for multiple comparisons.

A two-way ANOVA on *Gabra2* expression in the BNST of male mice revealed a significant genotype by treatment interaction [*F*_(1,27)_ = 4.693, *p* = 0.039], but no significant main effects of genotype [*F*_(1,27)_ = 0.093, *p* = 0.763] or treatment [*F*_(1,27)_ = 1.753, *p* = 0.197]. *Post hoc* analysis following the genotype by treatment interaction did not indicate any significant group differences (*p*’s > 0.05; Figure 3A). A two-way ANOVA on *Gabra2* expression in the CeA revealed a genotype by treatment interaction [*F*_(1,26)_ = 5.464, *p* = 0.027] and a main effect of treatment [*F*_(1,26)_ = 5.903, *p* = 0.022], but no main effect of genotype [*F*_(1,26)_ = 0.009, *p* = 0.927]. *Post hoc* analysis following the genotype by treatment interaction indicated that βE -/- males exhibited increased *Gabra2* expression following EtOH consumption, relative to basal conditions (*p* < 0.05; Figure 3B). A two-way ANOVA on *Gabra2* expression in the NAc revealed no significant main effect of genotype [*F*_(1,26)_ = 0.047, *p* = 0.830] or treatment [*F*_(1,26)_ = 0.3.271, *p* = 0.082], and no significant genotype by treatment interaction [*F*_(1,26)_ = 2.372, *p* = 0.136; Figure 3C]. A two-way ANOVA on *Gabra2* expression in the VTA revealed a genotype by treatment interaction [*F*_(1,26)_ = 4.798, *p* = 0.038], but no main effects of genotype [*F*_(1,26)_ = 0.687, *p* = 0.415] or treatment [*F*_(1,26)_ = 1.666, *p* = 0.208]. *Post hoc* analysis following the genotype by treatment interaction did not indicate any significant differences between groups (*p*’s > 0.05; Figure 3D).

## Discussion

This study supports the finding that genetic differences in βE expression affect binge-like EtOH consumption in a sex dependent manner (Nentwig et al., 2018), and further suggests that these effects involve modifications to GABAergic signaling in the limbic system. In female wildtype C57BL/6J mice, EtOH intake reduced *Gabra2* expression in multiple areas of the brain. In contrast, both wildtype and βE -/- males tended to increase expression of *Gabra2* mRNA after EtOH drinking. This finding is congruent with other studies using only males, which show that acute EtOH treatment increases *Gabra2* expression (Lindemeyer et al., 2017), while chronic alcohol exposure downregulates expression (Enoch et al., 2012; Jin et al., 2014; Forstera et al., 2016). Though we did not observe sex differences in EtOH intake in wildtypes animals as prior studies have reported (Tambour et al., 2008) this may be attributable to the number of drinking days used in various versions of the DID model. Sex differences in EtOH intake in the DID model are not always observed (Kaur et al., 2012; Nentwig et al., 2018) and appear more likely to emerge after several days to weeks of EtOH drinking, unlike the 4-day version used in the present study (Rhodes et al., 2005). Thus, the reductions in *Gabra2* expression in wildtype females may represent an adaptation that contributes to sex differences in binge EtOH intake as drinking progresses. Unlike wildtype counterparts, female βE -/- mice increased limbic expression of *Gabra2* mRNA following binge-like alcohol consumption. These mice also voluntarily consumed the most alcohol suggesting that the mechanisms responsible for sex differences in AUD development may involve βE interacting with GABA_A_ receptors in a sex-dependent manner.

The CeA is well-known for its role in chronic stress responses. It is responsible for converting emotionally relevant stimuli into behavioral and physiological responses, and it is highly interconnected with the NAc, BNST, and VTA (Gilpin et al., 2015). Previous studies have shown that EtOH increases GABA input onto the CeA which can disinhibit the BNST and VTA to reduce anxiety and stimulate reward, respectively (Leriche et al., 2008; Harrison et al., 2017). Interestingly, many studies examining the effects of alcohol on the CeA have been done exclusively in males. For example, in males, EtOH affects the activity of the CeA but not the BNST and *Gabra2* expression in the CeA is reduced in high anxiety or alcoholic subjects (Thiele et al., 1997; Jin et al., 2014; Skorzewska Lehner et al., 2015). Male rats innately have more GABAergic cells in the CeA compared to females (Ravenelle et al., 2014). More recently, studies have begun to include both males and females, and these seem to indicate that the effects of EtOH on the CeA in males is greater than in females (Logrip et al., 2017). The results of the present study further support the notion that the effects of alcohol on the CeA are different for males and females. More specifically, the greater effect of EtOH on CeA *Gabra2* expression in males is unmasked by the deficiency of βE expression, with EtOH consumption causing significant increases in *Gabra2* expression only in βE -/- males.

In females, EtOH affected *Gabra2* mRNA expression most dramatically in the BNST, an effect that was entirely dependent upon βE: the drug decreased *Gabra2* expression in wildtypes and while increasing it in βE -/- females. The BNST is an integral structure for the modulation of both the reward and stress neural circuitry. Most of the neurons in the BNST are GABAergic and activation of the BNST is generally anxiogenic (Ch’ng et al., 2018). Alcohol decreases the excitability of the BNST, which is crucial to the anxiolytic properties of EtOH (Leriche et al., 2008; Sharko et al., 2016). One of the ways that EtOH may be acting to reduce anxiety could be through increased expression of *Gabra2*. Stress and treatment with the stress neuropeptide, CRH, significantly increase the activity of the BNST neurons in females but not males (Sterrenburg et al., 2012; Babb et al., 2013; Salvatore et al., 2018). In addition, females innately have more CRH neurons in the BNST compared with males (Funabashi et al., 2004). The results of the present study provide additional support for the BNST as a critical mediator of the effects of EtOH in females, suggesting that the BNST is a critical node for the interaction of βE and sex in modulating the effects of EtOH on GABAergic signaling.

Data from a wide array of sources have suggested that females are inherently more vulnerable to stress-related disorders (Bale, 2009; Valentino and Bangasser, 2016). We previously showed that female βE -/- mice exhibit enhanced stress-sensitivity with hyperactivity of the HPA axis that can be ameliorated via binge-like EtOH consumption (Nentwig et al., 2018). While changes in mRNA expression do not necessarily translate to differences in functional receptor expression, in the present study stress-sensitive naive female βE -/- mice expressed significantly less *Gabra2* mRNA than βE +/+ mice in all of the brain regions examined. Similarly, data from both rats and mice demonstrated an association between lower baseline *Gabra2* expression and a high anxiety phenotype (Raud et al., 2009; Skorzewska Lehner et al., 2015). In addition, GABA_A_R agonist drugs like diazepam that reduce anxiety increase central *Gabra2* mRNA expression (Skorzewska Lehner et al., 2015). We see a similar effect here where EtOH intake, which has previously been shown to reduce the activity of the stress axis (Nentwig et al., 2018), increases *Gabra2* expression in female βE -/- mice. Therefore, our data and that of others supports a site-specific, sex-dependent inverse relationship between *Gabra2* expression and chronic upregulation of the HPA axis.

## Conclusion

Few preclinical studies have specifically examined the underlying mechanisms responsible for binge EtOH intake in females. The data presented here shed light on sexually dimorphic effects of voluntary drinking on GABAergic signaling that depend on βE expression. Along with previous studies, our results suggest an inverse correlation between *Gabra2* expression and anxiety, with subjects that have a lower baseline of *Gabra2* expression exhibiting an overly anxious phenotype and with *Gabra2* expression increases associated with significant anxiolytic responses. These data and others illustrate sex differences in central circuits mediating stress and reward that are responsible for the effects of EtOH on the brain, and perhaps provide a potential explanation for the increased proclivity of females to consume excessive quantities of EtOH, especially in the absence of βE.

## Author Contributions

ER, TN, and JG designed the study, performed the data analysis, and wrote the manuscript. TN, KL, and BC performed the behavioral data acquisition, and TN with the assistance of DW performed the qRT-PCR analysis.

## Conflict of Interest Statement

The authors declare that the research was conducted in the absence of any commercial or financial relationships that could be construed as a potential conflict of interest.
